# 3D analysis of human islet amyloid polypeptide crystalline structures in *Drosophila melanogaster*

**DOI:** 10.1371/journal.pone.0223456

**Published:** 2019-10-10

**Authors:** Ling Xie, Xiaohong Gu, Kenta Okamoto, Gunilla T. Westermark, Klaus Leifer

**Affiliations:** 1 Department of Engineering Sciences, Applied Materials Sciences, Uppsala University, Uppsala, Sweden; 2 Department of Medical Cell Biology, Uppsala University, Uppsala, Sweden; 3 Department of Biology Physics, Uppsala University, Uppsala, Sweden; Rijksuniversiteit Groningen, NETHERLANDS

## Abstract

Expression of the Alzheimer’s disease associated polypeptide Aβ42 and the human polypeptide hormon islet amyloid polypeptide (hIAPP) and the prohormone precursor (hproIAPP) in neurons of *Drosophila melanogaster* leads to the formation of protein aggregates in the fat body tissue surrounding the brain. We determined the structure of these membrane-encircled protein aggregates using transmission electron microscopy (TEM) and observed the dissolution of protein aggregates after starvation. Electron tomography (ET) as an extension of transmission electron microscopy revealed that these aggregates were comprised of granular subunits having a diameter of 20 nm aligned into highly ordered structures in all three dimensions. The three dimensional (3D) lattice of hIAPP granules were constructed of two unit cells, a body centered tetragonal (BCT) and a triclinic unit cell. A 5-fold twinned structure was observed consisting of the cyclic twinning of the BCT and triclinic unit cells. The interaction between the two nearest hIAPP granules in both unit cells is not only governed by the van der Waals forces and the dipole-dipole interaction but potentially also by filament-like structures that can connect the nearest neighbors. Hence, our 3D structural analysis provides novel insight into the aggregation process of hIAPP in the fat body tissue of *Drosophila melanogaster*.

## Introduction

To enable studies on protein aggregation and amyloid disease development [1], several research groups are using the GAL4-UAS system to drive cell or tissue specific expression of amyloid proteins in *Drosophila melanogaster* [2–4]. In the GAL4-UAS system the transcription activator protein Gal4 binds to the Upstream Activation Sequence (UAS) and activates gene transcription [5]. By selecting a particular Gal4 line, the protein expression can be directed to a particular population of cells or tissues. Earlier, we produced a *Drosophila melanogaster* line expressing human islet amyloid polypeptide (hIAPP). This is a 37 residues long polypeptide hormone that in humans is linked to islet amyloid formation which is present in almost all diagnosed with type 2 diabetes. hIAPP protein expression driven by the pan neuronal driver elav^C155^-Gal4 resulted in the formation of aggregates detected by two different amyloid specific dyes, Congo red and the fluorescent pentameric oligothiophene pFTAA. Further morphological analysis of these flies using transmission electron microscopy (TEM) revealed a second type of aggregates that was larger and highly ordered in fat body tissue surrounding the brain. In contrast, neither amyloid fibrils nor large well-ordered aggregates were detected in any of the five fly-lines expressing the non-amyloidogenic mouse IAPP (mIAPP) [6].

The hIAPP aggregates analyzed here were limited to the fat body tissue, which is a multifunctional organ important for the production and storage of lipids, proteins and carbohydrates, metabolic functions and detoxification [7, 8]. The basic fat body cell is a trichocyte, analogous to the vertebrate adipocyte, which is located in the hemocoel and in direct contact with the hemolymph [9]. Cells in fat body tissue of different types of insects resemble each other, as their trichocytes contain multiple small lipid droplets [10], different from mammalian white adipocytes which contain one or few large lipid droplets. After metamorphosis fat cells undergo cell death and reform in the adult fly from a distinct group of progenitor cells [11].

There are early TEM analyses describing pinocytotic uptake of nutrients from the hemolymph into the fat body [12] and peroxidase injection has been used to identify protein sequestration from the hemolymph and into storage in granules of the fat body [13]. In our earlier study [6], IAPP expression was driven to neurons by the pan neuronal driver elav^C155^-Gal4 and the peptide was transported over the blood brain barrier to the hemolymph and localizing to the fat body.

In studies on protein accumulation in the fat body of silk moth larvae, Tojo et al. described the presence of membrane enclosed granules with an amorphous outer region surrounding a dense core, often having a crystalline appearance [14]. However, this type of granule is not retained during maturation of larvae to adult insects.

Transthyretin (TTR) deposits as amyloid in many elderly people and lead to wild-type TTR-amyloidosis while mutations in the TTR gene can result in hereditary TTR-amyloidosis. In one fly model of TTR-amyloidosis, Pokrzywa et al. used GMR-Gal4 driver and found an accumulation of non-membrane-encircled nano-granules in both retinal and glial cells in flies expressing either the wild type TTR or the double mutant TTR (TTRV14N/V16E). While aggregates could not be detected in the brain fat body tissue of flies expressing two copies of TTRV14N/V16E, membrane-encircled aggregates were found in thoracic fat body cells. Independent of location, the formed aggregates consisted of highly ordered granules, with a diameter of 20 nm and were packed in hexagonal two dimensional (2D) arrangements. Such highly ordered protein aggregates located in the fat body could interfere with the normal functions of the tissue [3, 15], yet the three dimensional (3D) packing order of the TTR nano-granules was not investigated.

In mammalians, a well-known example of crystalline proteins is insulin which is stored in secretory granules [16] as dense crystals of insoluble hexameric insulin. Crystallization of insulin protects the molecules from further proteolytic processing [16] and prolongs the release of the hormone. However, the cellular mechanisms involved in the *in vivo* crystallization process have yet to be elucidated.

In the present study, we characterized the ultrastructure of three different non-amyloid protein aggregates, composed of hproIAPP, hIAPP and Aβ42, accumulated in *Drosophila melanogaster* using 2D and 3D imaging techniques on TEM. We used Bright field TEM (BFTEM) for measuring size and spacing of the subunit structure in the three protein aggregates. To test whether the protein aggregates constitute a storage form, 30 days old flies deprived of food for 24 and 48 hours were investigated by 2D BFTEM. Electron tomography on TEM was ultilized to reveal the 3D crystalline structure of protein granules in aggregates. The detailed packing orderings of protein granules were determined from reconstructed sub-volumes. A 2D-class averaging of protein granules was used to visualize the detailed structure present between two nearest protein granules and helps us to analyze the origin of the binding between the protein granules.

## Results

### 2D characterization and confirmation of IAPP in aggregates in the fat body

During the process of characterizing effects of hproIAPP and hIAPP expression in *Drosophila melanogaster* we observed accumulation of non-amyloid protein aggregates in fat body tissue surrounding the brain. Therefore, an extended electron microscopy analysis was conducted to elucidate the structure of these aggregates. To examine whether formation of this type of aggregates is limited to hproIAPP/hIAPP expression, flies expressing Aβ42 were included for the analysis.

**Fig 1:**
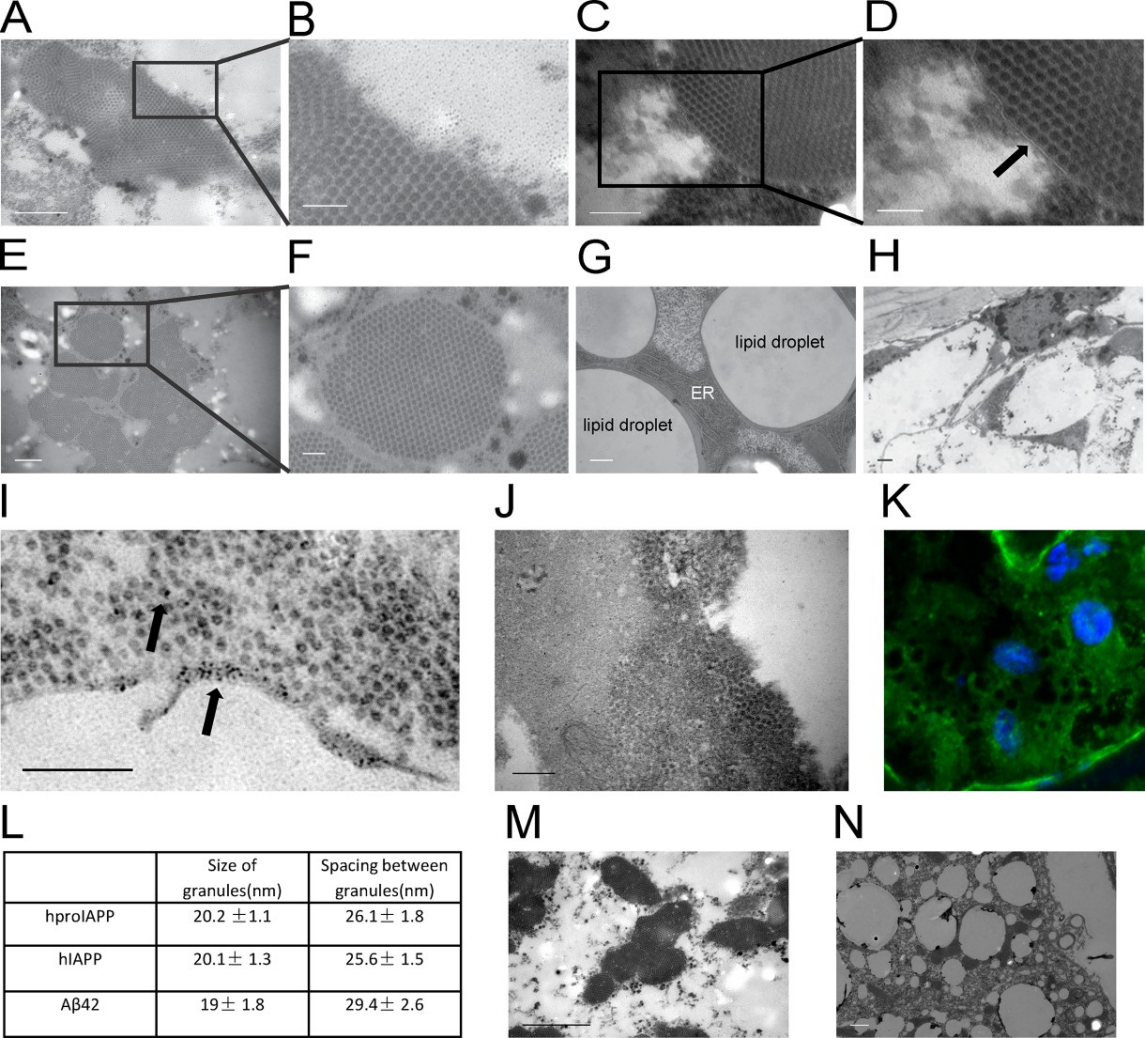
Ultra structural analysis and hIAPP immunoreactivity of aggregates and dissolution of aggregates after starvation in the fat body. Membrane encircled irregular shaped aggregates were detected in fat body cells of 30 days old flies with hproIAPP (A), hIAPP (C) and Aβ42 (E) expression driven by the pan neuronal driver elav^C155^-Gal4. Aggregates did not develop in flies expressing mIAPP (G) or in w^-^ flies (H). Black arrow (D) points to membrane structures. A minimum of 80 well separated subunits from hproIAPP (A), hIAPP (C) and Aβ42 (E) expressing flies were measured by drawing an intensity profile across each protein aggregate. Within an aggregate, smaller circular subunits were observed with a diameter of 20.2 ± 1.1 nm, 20.1 ± 1.3 nm and 19 ± 1.8 nm for hproIAPP, hIAPP and Aβ42, respectively (I). The spacing between two centers of the nearest hproIAPP, hIAPP and Aβ42 granules were 26.1 ± 1.8 nm, 25.6 ± 1.5 nm and 29.4 ± 2.6 nm, respectively (L). The results indicate a denser packing for hproIAPP and hIAPP aggregates compared to Aβ42 aggregates (p<0.001). Aggregate present in the fat body of a 30 days old fly was deteceted with IAPP-antiserum (I) and visualized with a secondary antibody labelled with 10 nm gold particles (indicated by arrows). In control material (J), the primary antiserum was omitted and no 10 nm gold particles could be detected. After immunolabelling tissues were embedded in Epon and counter stained with uranyl acetate and lead citrate. Formalin-methanol fixed heads from 30 days old flies were immunolabelled with anti-IAPP antibodies and reactivity was detected with secondary antibodies conjugated with Alexa647 demonstrate presence of IAPP (green) (K) in the cytoplasm of fat body cells. Nuclei are blue. 30 days old hproIAPP, hIAPP and Aβ42 female flies were starved with supplement of water for 24 and 48 hours. Flies alive at timepoints 24 and 48 hours were processed and investigated with TEM. Microscope analysis showed that all aggregates had disappeared from the fat body tissue already after 24 hours of starvation. M show presence of aggregates in the fat body of a proIAPP-expressing fly from the same crossing used for the starvation study while in (N), all aggregates have disappeared after 24 hours of starvation. Scale bar is 400nm in (A), (E), (G) and (H); 200nm in (C), (I) and (J); 100nm in (B), (D) and (F); and 1μm in (M) and (N).

In 50 nm ultrathin sections from flies expressing hproIAPP, hIAPP and Aβ42, similar irregular-shaped aggregates were observed in fat body tissue surrounding the brain (Fig 1A to 1F). Aggregates displayed differences in morphological appearance with sizes ranging from 0.5–5μm and were partially encircled by a membrane structure (Fig 1D). Five independent fly-lines with mIAPP expression driven by elav^C155^-Gal4 were analyzed for the presence of aggregates, but no such aggregates were detected in any fly from these lines (Fig 1G). This confirms that protein overexpression is not sufficient for aggregates to occur. As expected, aggregates were also absent in wild-type flies (Fig 1H).

Within an aggregate, smaller circular subunits were observed with a diameter of 20.2 ± 1.1 nm, 20.1 ± 1.3 nm and 19 ± 1.8 nm for hproIAPP, hIAPP and Aβ42, respectively (Fig 1L). The Aβ42 subunits were significantly smaller (p< 0.001) than the subunits present in hproIAPP and hIAPP aggregates. Spacing between subunits as measured from center to center of two nearest subunits was determined to 26.1 ± 1.8 nm for hproIAPP, 25.6 ± 1.5 nm for hIAPP and 29.4 ± 2.6 nm for Aβ42, and suggests a denser packing for hproIAPP and hIAPP aggregates when compared to packing in Aβ42 aggregates.

It was not possible to detect IAPP immunoreactivity in glutaraldehyde-fixed Epon or Lowicryl embedded tissue, therefore, immunolabelling was performed on frozen sections fixed in formalin. After incubation with an anti-IAPP antibody and a 10 nm gold-labeled detection antibody sections were post-fixed in glutaraldehyde and embedded in Epon. This procedure resulted in a slightly less-preserved morphology but it was still enough for the aggregates to be identified. Presence of 10 nm gold particles in the fat body tissue was limited to aggregates (Fig 1I). Gold particles were not detected when the primary antibody was excluded (Fig 1J). It is important to remember that in this procedure the immunelabeling is restricted to the top surface of the 10 μm thick frozen section, and that ultrathin sections are 40–60 nm thick. As a result, it is virtually impossible to simultaneously identify 10 nm gold particles throughout the surface of an aggregate.

In sections from fly heads subjected to immunolabelling with IAPP specific antiserum a reactivity was present in the cytoplasm of the fat body cells (Fig 1K).

### The dissolution of protein granules after 24 hours starvation

To determine whether aggregates represented a long-term storage-form or if they are utilized for short term metabolic purpose, 30 days old hproIAPP, hIAPP and Aβ42 female flies were food deprived but supplemented with water for 24 and 48 hours. Flies alive at timepoints 24 and 48 hours were processed and investigated with TEM. Microscope analysis showed that all aggregates had disappeared from the fat body tissue already after 24 hours of starvation (Fig 1N). Presence of aggregates prior to starvation was confirmed in flies from the three groups. The disappearance of aggregates points to a mechanism in *Drosophila melanogaste* for storage of aggregation-prone proteins into a form that can be metabolized when needed. Therefore, we conclude that two different types of aggregates are formed in *Drosophila melanogaster*, where one form represent amyloid detected by amyloid specific dyes Congo red and pFTAA as described earlier [6] and the second form described herein which represents a type of aggregates that accumulates in the fat body tissue and can be metabolized.

### Analysis of 5 fold twinning structure

What drives the aggregation is unknown but the rapid metabolization of aggregates during starvation points to a dynamic process. It is possible that formation of the highly ordered aggregates is influenced by the intracellular environment in the fat body cells. The spacing of two nearby hIAPP subunits is shorter than those of Aβ42, which indicates that hIAPP subunits have a more dense packing order than Aß42. This points to a stronger binding interaction between hIAPP subunits, thus more energy is required to dissolute the structure compared with Aβ42 aggregates. The more densely packed hIAPP subunits was selected for detailed 3D structural analysis.

**Fig 2:**
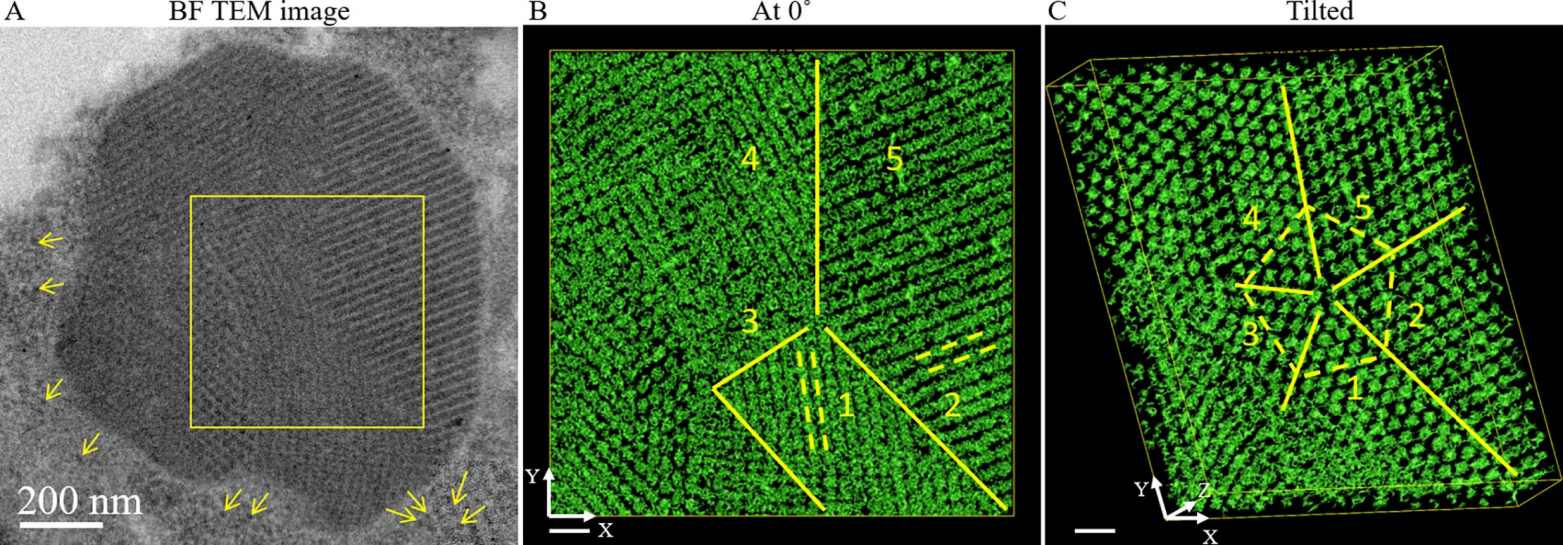
3D visualizations of hIAPP granules. (A) BF TEM image of one protein aggregate; the yellow arrows are pointing at the loosely distributed hIAPP granules surrounding the big protein aggregate. The 3D visualizations in (B) and (C) correspond to the yellow square in (A) and were produced by isosurface using IMOD. (B) The tomogram is positioned at 0°, and the hIAPP aggregates form lines indicated by yellow dashed lines in areas 1 and 2, separated by solid yellow lines. In (C), a 5-fold twinning was observed when the volume was tilted. The hIAPP granules appear as interconnected spheres and the yellow solid lines indicate the twinning boundaries. The dashed yellow lines in (C) indicate the (101) lattice planes of the BCT unit cell and the (110) lattice planes of the triclinic unit cell. Scale bar is 200 nm in (A) and 50 nm in (B) and (C). The side length of the volume in (B) and (C) are 600 nm (x) × 600 nm (y) × 85 nm (z).

The tilt series was acquired on the sample area shown in Fig 2A. In addition to the subunits that build up an aggregate, several individual subunits (see arrowheads in Fig 2A) were in close proximity to the highly organized structure. From these images alone, one cannot determine if the proximal subunits participated in growth of an aggregate or were just released from an aggregate. Additionally, the membrane, surrounding the aggregate was absent in these active areas. The segmented features in Fig 2B corresponded well with the dark features seen in Fig 2A, showing that reconstruction (S1 and S2 Movies) did not create significant artifacts in this projection at 0° tilt. Thus, we observed several regions with different types of subunit ordering in the protein aggregate of these two images. From this first observation, it can be concluded that the regions in the protein aggregate exhibited crystalline structures with a diameter of a few hundred nanometers. Moreover, in areas 1 and 2, the subunits appeared ordered along the lines as indicated by dashed lines (Fig 2B) which were not parallel. Instead these lines formed a 73° angle, indicating a grain boundary between these two ordered areas. By tilting the 3D volume, it can be determined that aggregates in Fig 2C were all crystalline and that subunits in the hIAPP aggregates exhibited an almost spherical shape (onwards referred to as protein granules). Evaluation of the entire region in Fig 2C reveal five grains with different orientations of the crystallites and a closer analysis allowed us to define these grain boundaries as twin boundaries. The angles formed by two adjacent twinning boundaries were 76° ± 1°, 70° ± 1°, 71° ± 1°, 73° ± 1°, and 69° ± 1°, respectively. Hence, the structure identified appears to be very similar to previously studied 5-fold twinning structures in Au nanoparticles [17].

### Analysis of the subunit ordering inside 5 fold twinning structure

**Fig 3:**
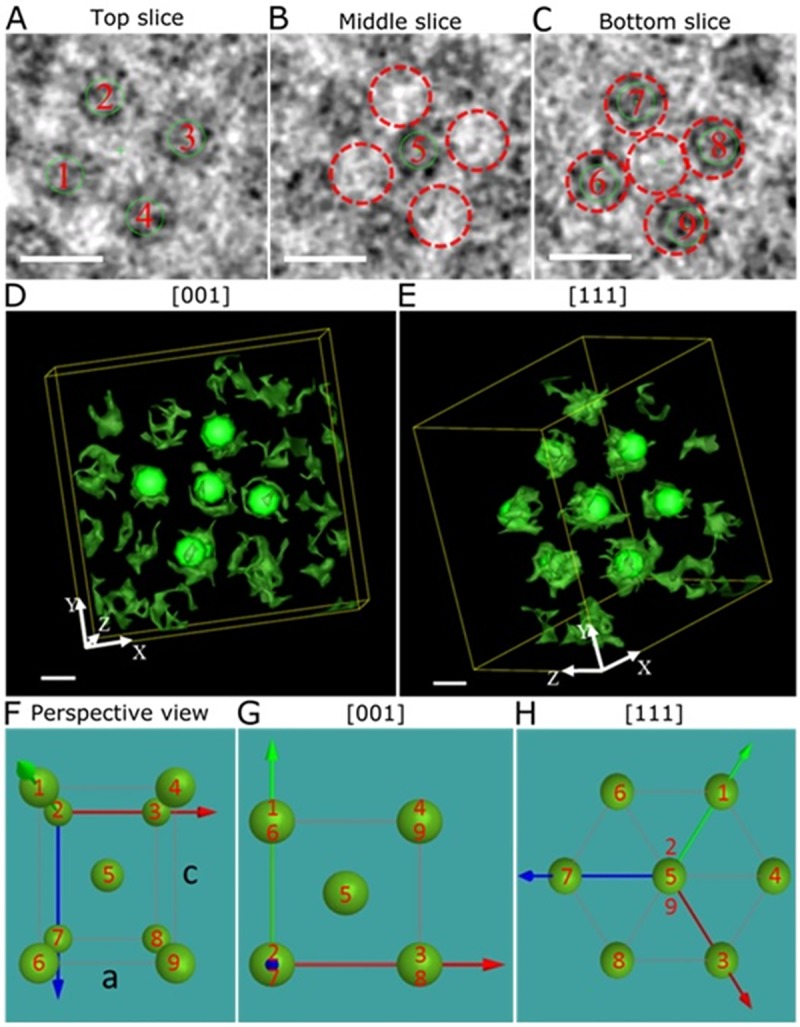
Body centered tetragonal structure formed by hIAPP granules. Three slices (each about 1.1 nm thick) were taken from area 1 of the reconstructed tomogram in Fig 3B. Green solid circles placed on the protein granules in (A), (B), and (C) correspond to the solid spherical markers in (D) and (E). In (D) and (E), the green markers highlight the structure of the BCT unit cell and help to identify the orientations of a unit cell. The distance between the slices was chosen so that each slice cuts through the center of a plane of the protein granules. The slices in (A), (B), and (C) are neighbored planes of the hIAPP granules. Red dashed circles in (B) and (C) indicate the projected hIAPP granules from (A) and (B), which were numbered 1–5. In (C), another four hIAPP granules were marked by solid green circles and are numbered 6–9. The distance between the slices in (A) and (C) was about 33 nm. 3D visualization of the same hIAPP aggregates after isosurface segmentation in IMOD. (D) shows the same projection as was used in (A-C). In (E), the image was tilted to a [111] zone axis of the BCT crystal. A perspective view of the BCT unit cell was shown in (F). The red, green, and blue arrows indicate the X, Y, and Z axis, respectively. (G) and (H) were the projections of the BCT unit cell along the [1] and the [111] zone axes, respectively. The distribution of the hIAPP granules numbered 1–9 in (A-C) is shown in (F-H). The volume shown in (D) and (E) has a side length of 150 nm (x) × 150 nm (y) × 50 nm (z). Scale bar is 20 nm in (A-E).

We resolved the crystalline structure of hIAPP granules in different regions of the 5-fold twinned structure by extracting sub-volumes from areas 1 and 2. We used three slices through area 1 (Fig 3A–3C; S3 and S4 Movies), to determine the crystalline order. Nine green markers, positioned approximately in the center of the granules in these three slices, constitute the unit cell of the body centered tetragonal (BCT) structure with lattice parameters a = 27.9 ± 1.1 nm and c = 33 ± 1.1 nm. The three slices were acquired along the [1] structural zone axis (Fig 3D). Protein granules present in the top and bottom slices were not perfectly aligned (Fig 3A and 3C). However, small deviations from a perfect BCT lattice are expected due to weak van der Waals interactions between granules [18, 19]. Further evidence of a BCT unit cell can be found after tilting this structure along the diagonal line by 50°, where the [111] zone axis of the same unit cell is identified (Fig 3E). In the BCT unit cell drawn in Fig 3F, there are eight spheres sitting on the corners that correspond to hIAPP granules numbered 1–4 and 6–9 in Fig 3A and 3C, and one sphere positioned at the center corresponding to number 5 marked in Fig 3B. The spheres in Fig 3G and 3H containing two or three numbers indicate an overlap of hIAPP granules along the corresponding zone axis and provide projections that are closely correlated to the patterns seen in Fig 3D and 3E, when the BCT unit cell is oriented to the [1] and [111] zone axes.

**Fig 4:**
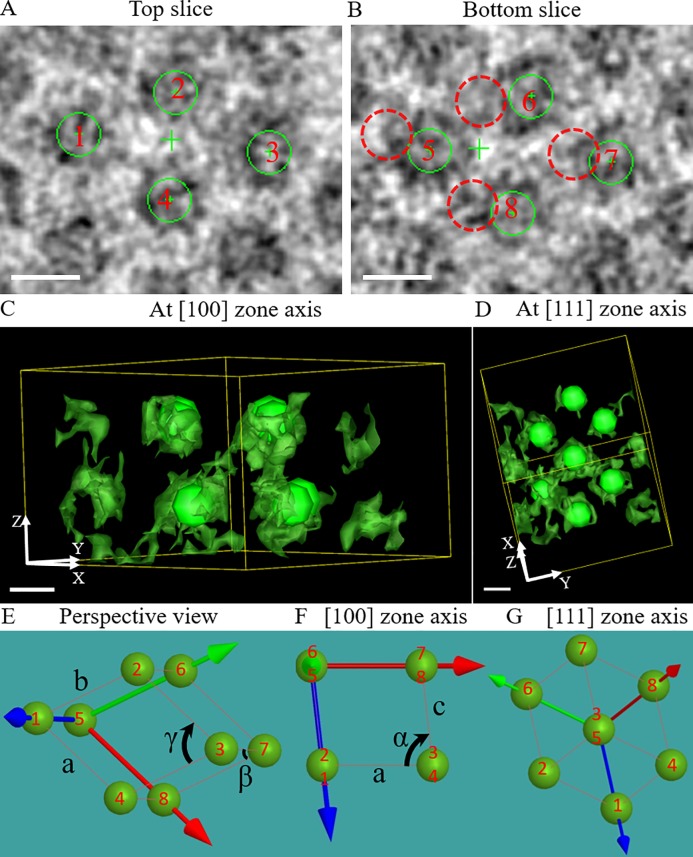
Triclinic structure formed by hIAPP granules. Two slices (each about 1.1 nm thick) were taken from area 2 of the reconstructed tomogram in Fig 3B. Green solid circles placed on the protein granules in (A) and (B) correspond to solid spherical markers in (C) and (D). The green markers highlight the structure of the triclinic unit cell and help to identify the orientations of a unit cell. The distance between slices was chosen so that each slice cuts through the center of a plane of protein granules, and the slices in (A) and (B) are the neighboring planes of the protein granules. Red dashed circles in (B) indicate the projected position of the hIAPP granules in (A), which were numbered 1–4. In (B), another four hIAPP granules were marked by solid green circles and numbered 5–8. The distance between the slices in (A) and (B) is about 20 nm. 3D visualization of the hIAPP granules was done by isosurface segmentation using IMOD. A perspective view of the triclinic structure was shown in (E); the green, red, and blue arrows indicate the X, Y, and Z axis, respectively. (F) and (G) are the projections of the triclinic unit cell along the [100] and the [111] zone axis, respectively. The distribution of the hIAPP granules numbered 1–8 in (A) and (B) was represented by green spheres in (E-G). Volumes in (C) and (D) have a side length of 100 nm (x) × 100 nm (y) × 70 nm (z). Scale bar is 20 nm in (A-D).

Analysis of the extracted area 2 reveal a differing crystalline structure where protein granules were ordered into triclinic structure, with lattice parameters a = 28.4 ± 1.1 nm, b = 26.5 ± 1.1 nm, c = 24.2 ± 2.1 nm, α = 78° ± 1°, β = 66° ± 1°, and γ = 61° ± 1° (Fig 4A and 4B; S5 and S6 Movies). In Fig 4C, a projection along the [100] zone axis of this structure can be observed and by rotating the 3D volume, the structure is visualized along the [111] zone axis of the same unit cell (Fig 4D). In contrast to a BCT unit cell, the angles (α, β, γ) are not equal to 90°, a defining characteristic of a triclinic unit cell structure. Eight green spheres sitting on the corners correspond to hIAPP granules numbered 1–8 (Fig 4A and 4B). Unlike the BCT unit cell, a triclinic unit cell does not have a centrally located protein granule and the projections of the triclinic unit cell oriented to the [100] and [111] zone axes displayed (Fig 4F and 4G) are in good agreement with the observation from Fig 4C and 4D.

Additionally, volumes extracted from areas 3, 4 and 5 were analyzed. We observed that areas 1 and 4 are composed of BCT unit cells, whereas 2, 3 and 5 are composed of triclinic unit cells. The entire 5-fold twinned crystalline structure contained cyclic twinning of the BCT and triclinic unit cells, except for the twinning between areas 2 and 3 which was generated by the same triclinic unit cell.

### 2D-class averaging of protein granules

**Fig 5:**
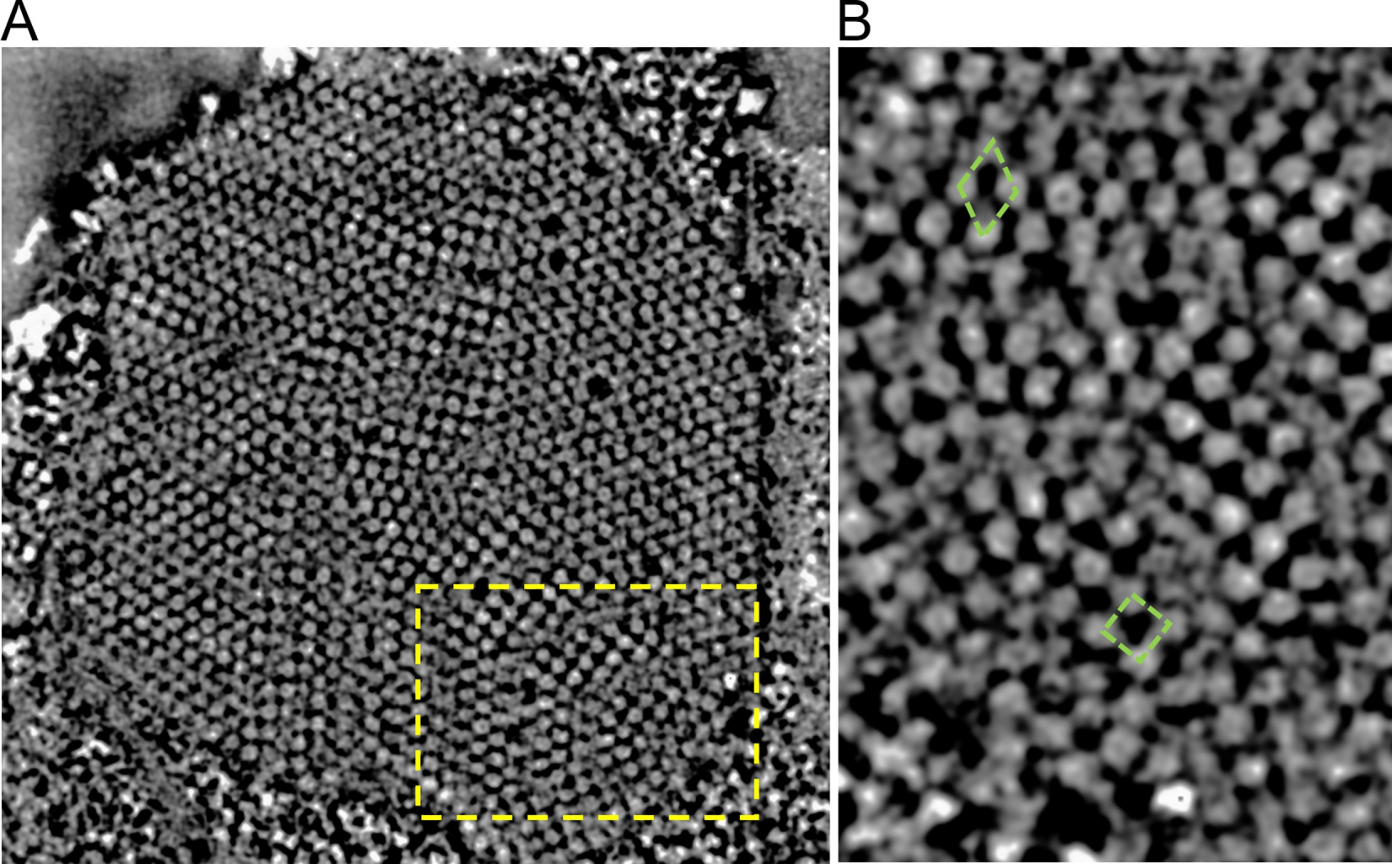
Linkers are observed from 3D tomogram. (A) 20 nm slice extracted from reconstructed tomogram and projected onto XY plane. (B) Enlarged image of the yellow dash line marked area in (A). Two green dash line marked areas in (B) are the (001) planes of triclinic (on the top) and BCT (at the bottom) unit cell, respectively.

From the above sections, we learned that hIAPP granules have a strong tendency to form BCT and triclinic unit cells instead of the other twelve Bravais lattices. Accordingly, it is surprising that the granules are not crystallized in the densest stacking mode consisting of face centered cubic (FCC) or hexagonal close packing (HCP) structures. Packing of granules can be governed by van der Waals forces and dipole-dipole interaction, but also potentially by physical structures present between two nearest granules. We extracted a 20 nm layer along the XY plane from the center region of the reconstructed tomogram (in Fig 5A). The contrast is inverted in the extracted layers from the raw reconstructed slices, therefore, the bright dot-like contrast shown in Fig 5 indicates the protein granules. Since the layer was extracted along XY plane, most of the protein granules are sitting at the (001) lattice planes of triclinic or BCT unit cells. These protein granules distributed at (001) lattice plane have nearest distance in both unit cell and are connected by thin filaments (as shown in Fig 5B), hereafter referred to as “linkers”. It cannot be excluded that the sample preparation procedure generated the detected linkers, but they do occur in an organized manner. Accordingly, further analysis was performed to investigate the preferred orientations of the linkers. We selected 30 protein granules along the (001) lattice planes in BCT structures and the (001) lattice planes in triclinic structures and used the 2D-class averaging to average the contrasts of the selected particles generating the classifications. From the class-averaged particles, we clearly discerned the protein granules were interconnected by linkers. When a translational alignment of boxed protein granules was applied in the 2D classification, the angle formed by two linkers was conserved in class-averaged particles.

The average position of the linkers was analyzed with respect to the crystalline direction of granules (S2 Fig). We found that the protein granules selected from the (001) lattice plane of the BCT structure, which are connected by the linkers (indicated by blue dashed lines in S2A and S2B Fig) formed angles of 90° and 180°. Similarly, we selected protein granules distributed in the (001) lattice planes (S2C Fig) from the triclinic structure. Similar to the BCT structure, we found that the linkers existed between every two neighbored granules in both the (001) and lattice planes (S2D Fig).

## Discussion

ET analysis on TEM provides detailed 3D structure at a nanometer scale [19, 20]. Here we applied electron tomography to study the 3D distribution of macromolecular aggregates formed in fat body cells of the *Drosophila melanogaster*. These highly ordered non-amyloid-like aggregates were detected during the characterization of the *Drosophila melanogaster* expressing the hIAPP [6]. The fat body tissue is unique to insects, and it is well- known that this tissue can store large amounts of proteins used for nutrition during developing stages when no feeding occurs [21–23]. Previously, there have been indications that protein can accumulate in the adult fly [24], but aggregates with an ultra-structure in all three dimensions have not been described.

Our electron tomography results show that hIAPP aggregates formed subunits similar to spheres, (granules), and that these granules precipitated into BCT and triclinic structures,. Intriguingly, further analysis of these 3D structures elucidated a 5-fold twinned structure formed by approximately two thousand protein granules. As far as we know, this is the first time that 5-fold twinned structures generated by biological materials have been identified while in material science, 5-fold twinned structures have been observed in both Au [25] and Ag [26] nanoparticles (NPs). The Tricorn protease was shown to exist in a hollow 5-fold symmetric structure [27], however, herein, we present a 5-fold twinned structure with a complete space filling. In the examined samples, five-fold twinning was observed in multiple aggregates, but the in depth 3D analysis was performed on a single hIAPP aggregate.

When a 5-fold twinned structure develops via accumulation of individual entities, the growth can be either nucleation based or growth mediated [17]. In the latter case, several pre-existing structures, such as tetrahedral structures, can aggregate to form multiple twinned NPs. During nucleation-based growth, a small crystalline nucleus forms in the initial state, and then crystalline packing of the protein granules is complemented via layer-by-layer growth in all three dimensions. Presence of individual granules in close proximity to the larger aggregate in our studies suggests our model uses nucleation-based crystalline growth. However, in our case the nucleation-based growth model alone cannot completely explain the crystalline order formed. For nucleation-based growth, dense packing order of either FCC or the HCP structures would be expected, instead of the BCT and the triclinic structures observed. The binding energy of two nearby globular protein aggregates can be modeled by Hamaker two-body method (S1 Data File) and it is on the order of 0.02 eV. On the one hand the small binding energy could originate from the phase transformations and the different crystalline phases and crystalline lattice defects observed in this work. On the other hand, because these are isotropic and weak interactions, they could underlie dense packed stacking orders. However, we only discovered BCT and triclinic crystalline structures implying that granules use non-isotropic or directional binding forces to their neighbor granules to form these non-densely packed structures.

The observation of linkers between two nearest neighbored protein granules indicates that directional interactions between nearest neighbored granules may play a role in structure formation of protein aggregates. However, from this study, it is not clear that such non-isotropic linking can lead to the formation of observed crystalline packing of protein granules. The analysis of the linkers was performed on material prepared for electron microscopy, therefore it cannot be ruled out that the linkers identified between protein granules have arisen as a result of the preparation. However, their occurrence could be responsible for the packing of granules into BCT and triclinic structures instead of the more dense FCC and HCP structures.

The crystalline arrangement facilitates a denser packing of the protein granules, but the significance of these aggregates is not obvious. The amount of larger electron dense aggregates depicted in Fig 1 could be extensive and occupy a substantial part of the fat body tissue affecting functions of the tissue. The aggregates were often enclosed by a membrane. However, this membrane was absent in areas rich in individual granules, implying a potential ongoing growth of the protein aggregates. The multifunctional fat body tissue can be used for the storage of protein and also for detoxification. To determine if aggregates can be recycled, 30 days old hproIAPP, hIAPP and Aβ female flies were starved for 24 hours and 48 hours. Investigation after starvation showed a complete disappearance of aggregates (Fig 1) and support our theory that formation of aggregates represents an important biological function. Amyloid fibrils occur through the formation of aggregates with distinct structures. In a fibril formation study using a 14-residue long Aβ-like peptide, a crystalline structure was identified which occurred for a few minutes early in the process, to finally be replaced by mature amyloid fibrils [28]. The transient structure called nanocrystals had substructure size of 50 Å compared to 20 nm of the granules described here.

It is interesting that proteins with high aggregation potential can adopt a highly ordered non-fibrillar structure. There are no reports that similar aggregates occur in other species e.g. mammals, but that does not exclude that highly ordered aggregates could develop. However, it is more likely that a unique mechanism exists that is either general to insects or limited to Drosopila melanogaster. Analysis of gene expression and protein synthesis in fat body tissue from proIAPP, hIAPP and Aβ 42 expressing flies will help us identify possible candidate proteins involved in the process. Thereafter, the knowledge can be used to determine if similar proteins are present in mammals and whether a similar aggregation may be possible.

In summary, this observation of morphology, along with the structural analysis, shows that aggregates develop dynamically during the life of the *Drosophila melanogaster*.

## Methods

### *Drosophila melanogaster* strains

The transgene fly lines UAS-hIAPP and UAS-mIAPP cloned with human proinsulin secretion signal peptide and UAS-hpreproIAPP have been described [6], and the transgene fly line UAS-human Aβ42 cloned with a Drosophila necrotic gene secretion signal peptide was kindly provided by D.C. Crowther, Cambridge, UK [29]. The Gal4 driver line, P{w[+mW.hs] = GawB}elav[C155], was obtained from the Bloomington *Drosophila* Stock Center, Indiana University.

Transgene expression was achieved by crossing the UAS-transgene fly line with the elav^C155^-Gal4 driver line [5]. Negative control flies (w^-^) were generated by crossing the w^1118^ flies with the elav^C155^-Gal4 flies to exclude any effects of the Gal4 protein expression. Flies were cultured on standard fly food and maintained in a programmed chamber (KBWF 240, Binder, Germany) with 26°C and 70% humidity at a 12/12-hour light/dark cycle. Flies were transferred to fresh food vials every other day. Female flies, aged 30 days, were used in the morphology study.

### Transmission Electron Microscopy (TEM)

Aged flies were sedated with CO_2_ and carefully decapitated. The heads were fixed in 2.5% glutaraldehyde in 4.3 mM Na_2_HPO_4_, 1.4 mM KH_2_PO_4_, 2.7 mM KCL, and 137 mM NaCl, buffer (PBS), pH 7.2 for 24 hours followed by post-fixation in 1% OsO_4,_ dehydration and embedding in Epon (Agar Scientific, Essex, United Kingdom). Ultrathin sections including part of the brain and frontal fat body were placed on formvar coated copper grids and contrasted with 5% uranyl acetate in water and with a lead citrate solution. The material was studied at 75 kV in a Hitachi H-7100 TEM (Hitachi, Tokyo, Japan). Images were taken with a digital camera (Gatan, Pleasanton, U.S.A.) and processed using the Digital Micrograph software package (version 2.30.542.0, Gatan). The size of each protein granule and the spacing between the two adjacent protein granules were measured from the intensity profile on the Digital Micrograph, Gatan (version 2.30.542.0). The unit cells of crystalline structures were sketched using the electron microscopy analysis software, JEMs for Fig 3 and Fig 4 (version 3.8431U2012) and VESTA [30](version 3.3.2) for Fig 5. The lattice parameters chosen to set up the unit cell were scaled to the measured lattice parameters from the reconstructed tomograms.

### Starvation of the flies

30 days old hproIAPP, hIAPP and Aβ female flies were kept in empty food tube with water supplements. Flies were decapitated after 24 hours and 48 hours and their heads were studied according to the TEM procedure.

### Immunofluorescense

Cryo-sections from 30 days old flies were placed on slides and fixed in 10% neutral buffered formalin for 10 minutes and thereafter ice cold methanol for 10 minutes. Incubation with antiIAPP antibodies diluted 1:100 was carried out over night at room temperature. Reactivity was visualized with antirabbit Alexa647 antibodies diluted 1:1000 and nuclei stained with DAPI. The sections were analysed in a Zeiss LSM780 confocal microscope equipped with argon 488-nm and HeNe 543-nm lasers (Zeiss, Oberkochen, Germany).

### Immuno electron microscopy

Snap-frozen heads from 30-day-old hIAPP flies were cut 10 μm thick, placed on slides and fixed in 10% buffered neutral formalin for 10 minutes. Heads from non-transgenic flies were used as negative control. The tissue was rinsed in 50 mM trisHCl buffer with 150 mM NaCl, (TBS) followed by blocking in 3% bovine serum albumin (BSA) in TBS for 1 hour at room temperature (RT). The tissue was incubated with primary antiserum specific for IAPP, diluted 1: 200 in 1% albumin in TBS at RT overnight. After rinsing with TBS, sections were incubated with a secondary antibody labeled with 10 nm gold particles for 2 hours at RT, followed by rinsing in TBS and distilled water. Sections were carefully removed from the slide and fixed in 2.5% glutaraldehyde in PBS for 24 hours followed by post-fixation in 1% OsO_4,_ for 2 minutes, then dehydrated and embedded in Epon. Ultrathin sections were placed on formvar coated copper grids and contrasted with 5% uranyl acetate in water and a lead citrate solution. Sections were investigated as describe above.

### Electron tomography

To allow for an alignment of individual images in a tilt series, the grid was immersed in 10 nm gold conjugated goat anti-mouse IgG (British Biocell International, Cardiff, U.K.) diluted 1: 500 with milliQ water for 5 minutes, rinsed in milliQ water, and air dried prior to analysis. Electron tomography was carried out using a FEI Tecnai F30-ST field emission TEM, equipped with a CCD camera with 2048×2048 pixels. To minimize the missing wedge effect, a double tilt series was acquired using a Fischione model 2020 tomography sample holder. The tilt series was acquired from ±60˚ with 2˚ increments with bright field (BF) TEM. The focusing condition is controlled by looking at the Fresnel fringes of the gold nanoparticles and the Fourier transform of the image. Alignment and reconstruction of the image stack were carried out using the IMOD software package (version 4.7.11) [31, 32]. During the data acquisition, the defocus was controlled in a range of 800 nm ± 20 nm. The images obtained under this defocusing condition generate adequate phase contrast for the subsequent alignment and reconstruction. Also, the first zero of the contrast transfer function under this defocusing condition gives a spatial resolution of 1.3 nm in the XY plane and is sufficient to resolve 3D structural information above 3 nm. This value is calculated by taking into consideration the elongation factor of 1.44 (for a dual axis data acquisition) in the Z direction [33], then the final resolution R in the Z direction is determined by 1.3 nm multiplied by the elongation factor plus the alignment errors (about 2 voxels, ~ 1.1 nm), where R is equal to 3 nm. To avoid irradiation damage onto the specimen at 300kV, the electron dose is less than 1,500 electrons per nm^2^ [34]. The specimen was irradiated under the electron beam for 30 minutes before the data acquisition.

### 2D-class averaging of the protein granules

The thickness of the layer was adjusted to about 20 nm, which is equal to the diameter of the protein granule particles. Thus, the whole volume of the particle was embedded in the layer. The contrast was inverted in the extracted layers from the raw reconstructed slices, therefore, the bright dots like contrast shown in Fig 5 indicates the protein granules. Layers were prepared along the (001) and the (101) lattice planes in the BCT crystals and the (100) and the (001) lattice planes in the triclinic crystal using the “slicer” tool in IMOD. The particle images were extracted from the projections of the 3D tomograms. EMAN2 (e2boxer.py) was used for selecting 30 individual particles both in BCT and triclinic unit cells from the 34 projections in the reconstructed 3D tomogram. The 2D class-averaged particle images were calculated by translational alignment in MSA (Multivariate statistical analysis)-based reference-free classification using EMAN2 (e2classavearge.py) [35]. After eight iterative steps of the 2D-classification, the final three class-averaged particle images were generated from both BCT and triclinic unit cells. The class-averaged particle images were visualized by Chimera software [36].

### Statistical analysis

One-way ANOVA with Dunnett’s multiple comparisons test was performed using GraphPad Prism Version 6.01 (GraphPad software, La jolla, CA). P <0.05 was considered statistically significant.

## Supporting information

S1 Fig Bending effect and vacancies of hIAPP granules along the Z direction. 3D visualization of hIAPP aggregates by performing isosurface segmentation in IMOD. Red dashed lines indicate the bended distribution of hIAPP protein granules in the Z direction. Red dashed circles indicate absence of protein granules. (A) The centre slice from reconstructed tomogram, (B) 50 nm (x) × 650 nm (y) × 150 nm (z) volume. The scale bar in (A) is 100 nm and in (B) is 20 nm. A video was made to show the YZ slices moving from slice No. 300 to No. 1000 along X axis direction in S7 Movie.
(TIF)

S2 Fig 2D-class averaging analysis of protein granules. (A) and (C) show the layers parallel to the (001) and the (101) lattice planes that were cut from the BCT unit cell. (B) and (D) are the three averaged classes from the selected particles from (A) and (C). The dashed blue lines indicate the orientations of the linkers between the two nearest protein granules.
(TIF)

S1 Data File The binding energy between two nearest protein granules was calculated based on the Hamaker two-body method. (DOCX)

S1 Discussion Number density of hIAPP granules and the structural imperfections. (DOCX)

S1 Movie Z walk through the reconstructed tomogram of one hIAPP protein aggregation. The tomogram was reconstructed from a ~85 nm thick sample and divided into 77 slices. The video was created using ImageJ/Fiji function.
(AVI)

S2 Movie Surface rendered tomogram as shown in Fig 2B tilting around X and Y axis for 180°. The video was created using ImageJ/Fiji function.
(AVI)

S3 Movie Z walk through the reconstructed tomogram of BCT unit cell. The tomogram was reconstructed from a ~50 nm thick sample and divided into 45 slices. The video was created using ImageJ/Fiji function.
(AVI)

S4 Movie Surface rendered BCT structure tilting around X and Y axis for 180°. The green markers highlight the structure of BCT unit cell and help to identify orientations of the unit cell. The video was created using ImageJ/Fiji function.
(AVI)

S5 Movie Z walk through the reconstructed tomogram of triclinic unit cell. The tomogram was reconstructed from a ~45 nm thick sample and divided into 41 slices. The video was created using ImageJ/Fiji function.
(AVI)

S6 Movie Surface rendered triclinic structure tilting around X and Y axis for 180°. The green markers highlight the structure of triclinic unit cell and help to identify orientations of the unit cell. The video was created using ImageJ/Fiji function.
(AVI)

S7 Movie X walk of YZ plane through the reconstructed tomogram. The video was created using ImageJ/Fiji function.
(AVI)

## References

[1] Benson MD, Buxbaum JN, Eisenberg DS, Merlini G, Saraiva MJM, Sekijima Y, et al Amyloid nomenclature 2018: recommendations by the International Society of Amyloidosis (ISA) nomenclature committee. Amyloid. 2018;25:215–219. 10.1080/13506129.2018.1549825 30614283

[2] Crowther DC, Page R, Chandraratna D, Lomas DA. A Drosophila model of Alzheimer's disease. Methods Enzymol. 2006;412:234–255. 10.1016/S0076-6879(06)12015-7 17046662

[3] Pokrzywa M, Dacklin I, Hultmark D, Lundgren E. Misfolded transthyretin causes behavioral changes in a Drosophila model for transthyretin-associated amyloidosis. Eur J Neurosci. 2007;26:913–924. 10.1111/j.1460-9568.2007.05728.x 17714186

[4] Berg I, Thor S, Hammarstrom P. Modeling familial amyloidotic polyneuropathy (Transthyretin V30M) in Drosophila melanogaster. Neurodegener Dis. 2009;6:127–138. 10.1159/000213761 19372706

[5] Brand AH, Perrimon N. Targeted gene expression as a means of altering cell fates and generating dominant phenotypes. Development. 1993;118:401–145. 8223268 10.1242/dev.118.2.401

[6] Schultz SW, Nilsson KP, Westermark GT. Drosophila melanogaster as a model system for studies of islet amyloid polypeptide aggregation. PLoS One. 2011;6:e20221 10.1371/journal.pone.0020221 21695120 PMC3114789

[7] Arrese EL, Soulages JL. Insect fat body: energy, metabolism, and regulation. Ann Rev Entomol. 2010;55:207–225. 19725772 10.1146/annurev-ento-112408-085356 PMC3075550

[8] Haunerland N, Shirk P. Regional and functional differentiation in the insect fatbody. Ann Rev Entomol. 1995;40:121–145.

[9] Chapman R. Fat Body In: Simpson S, Douglas A, editors. The Insects: Structure and Function. Cambridge University Press; 2013 p. 132–144.

[10] Butterworth FM, Emerson L, Rasch EM. Maturation and degeneration of the fat body in the Drosophila larva and pupa as revealed by morphometric analysis. Tissue Cell. 1988;20:255–268. 10.1016/0040-8166(88)90047-x 3136556

[11] Hoshizaki DK, Lunz R, Ghosh M, Johnson W. Identification of fat-cell enhancer activity in Drosophila melanogaster using P-element enhancer traps. Genome. 1995;38:497–506. 10.1139/g95-065 7557362

[12] Gaudecker B. Über den Formwechsel einiger Zellorganelle bei der Bildung der Reservestoffe im Fettkörper von Drosophila-Larven. Z Zellforscnung. 1963;61:56–95. 14095100

[13] Locke M, Collins JV. Protein uptake into multivesicular bodies and storage granules in the fat body of an insect. J Cell Biol. 1968;36:453–483. 10.1083/jcb.36.3.453 5645544 PMC2107381

[14] Tojo S, Betchaku T, Ziccardi VJ, Wyatt GR. Fat body protein granules and storage proteins in the silkmoth, Hyalophora cecropia. J Cell Biol. 1978;78:823–838. 10.1083/jcb.78.3.823 701361 PMC2110208

[15] Pokrzywa M, Dacklin I, Vestling M, Hultmark D, Lundgren E, Cantera R. Uptake of aggregating transthyretin by fat body in a Drosophila model for TTR-associated amyloidosis. PLoS One. 2010;5:e14343 10.1371/journal.pone.0014343 21179560 PMC3002944

[16] Doye J, Poon W. Protein crystallisation in vivo. Curr Opin Colloid Interface Sci. 2006;11:40–46.

[17] Hofmeister H. Fivefoled twinned nanoparticles In: Nalwa S, editor. Encyclopedia of Nanoscience and Nanotechnology 3. Valencia: American Scientific Publishers; 2004 p. 432–452.

[18] Nir S, Andersen M. Van der Waals interactions between cell surfaces. J Membr Biol. 1977;31:1–18. 10.1007/bf01869396 839528

[19] Olins DE, Olins AL, Levy HA, Durfee RC, Margle SM, Tinnel EP, et al Electron microscope tomography: transcription in three dimensions. Science. 1983;220:498–500. 10.1126/science.6836293 6836293

[20] Skoglund U, Andersson K, Strandberg B, Daneholt B. Three-dimensional structure of a specific pre-messenger RNP particle established by electron microscope tomography. Nature. 1986;319:560–564. 10.1038/319560a0 3945344

[21] Roma GC, Bueno OC, Camargo-Mathias MI. Morpho-physiological analysis of the insect fat body: a review. Micron. 2010;41:395–401. 10.1016/j.micron.2009.12.007 20206534

[22] Rizki TM, Rizki RM. Larval adipose tissue of homoeotic bithorax mutants of Drosophila. Dev Biol. 1978;65:476–482. 10.1016/0012-1606(78)90042-8 98371

[23] Raman C. Electron microscopy and immunogold labelling analysis of smart nanoparticles in insects In: Méndez-Vilas A, editor. Current microscopy contributions to advances in science and technology. 1 Spain: Formatex publisher; 2012 p. 168–178.

[24] Butterworth FM, Bownes M, Burde VS. Genetically modified yolk proteins precipitate in the adult Drosophila fat body. J Cell Biol. 1991;112:727–737. 10.1083/jcb.112.4.727 1899669 PMC2288856

[25] Neumann W, Komrska J, Hofmeister H, Heydenreich J. Interpretation of the shape of electron diffraction spots from small polyhedral crystals by means of the crystal shape amplitude. Acta Crystallographica Section A. 1988;44:890–897.

[26] Smit J, Ogbum F, Bechtoldt C. Multiple Twin Structures in Electrodeposited Silver Dendrites. J Electrochem Soc. 1968;115:371–374.

[27] Walz J, Tamura T, Tamura N, Grimm R, Baumeister W, Koster AJ. Tricorn protease exists as an icosahedral supermolecule in vivo. Mol Cell. 1997;1:59–65. 10.1016/s1097-2765(00)80007-6 9659903

[28] Otzen DE, Oliveberg M. Transient formation of nano-crystalline structures during fibrillation of an Abeta-like peptide. Protein Sci. 2004;13:1417–1421. 10.1110/ps.03538904 15096642 PMC2286749

[29] Crowther DC, Kinghorn KJ, Miranda E, Page R, Curry JA, Duthie FA, et al Intraneuronal Abeta, non-amyloid aggregates and neurodegeneration in a Drosophila model of Alzheimer's disease. Neuroscience. 2005;132:123–135. 10.1016/j.neuroscience.2004.12.025 15780472

[30] Momma K, Izumi F. VESTA 3 for three-dimensional visualization of crystal, volumetric and morphology data. J Appl Crystallogr 2011;44:1272–1276.

[31] Mastronarde DN. Automated electron microscope tomography using robust prediction of specimen movements. J Struct Biol. 2005;152:36–51. 10.1016/j.jsb.2005.07.007 16182563

[32] Kremer JR, Mastronarde DN, McIntosh JR. Computer visualization of three-dimensional image data using IMOD. J Struct Biol. 1996;116:71–76. 10.1006/jsbi.1996.0013 8742726

[33] Mastronarde DN. Dual-axis tomography: An approach with alignment methods that preserve resolution. J Struct Biol. 1997;120:343–352. 10.1006/jsbi.1997.3919 9441937

[34] Norlin N, Hellberg M, Filippov A, Sousa AA, Grobner G, Leapman RD, et al Aggregation and fibril morphology of the Arctic mutation of Alzheimer's A beta peptide by CD, TEM, STEM and in situ AFM. J Struct Biol. 2012;180:174–189. 10.1016/j.jsb.2012.06.010 22750418 PMC3466396

[35] Tang G, Peng L, Baldwin PR, Mann DS, Jiang W, Rees I, et al EMAN2: An extensible image processing suite for electron microscopy. J Struct Biol. 2007;157:38–46. 10.1016/j.jsb.2006.05.009 16859925

[36] Pettersen EF, Goddard TD, Huang CC, Couch GS, Greenblatt DM, Meng EC, et al UCSF chimera—A visualization system for exploratory research and analysis. J Comput Chem. 2004;25:1605–1612. 10.1002/jcc.20084 15264254

