# Supplementary figures and images for: 3D analysis of human islet amyloid polypeptide crystalline structures in Drosophila melanogaster
Source: PLoS One. 2019 Oct 10;14(10):e0223456. doi: 10.1371/journal.pone.0223456 (PMC6786548; doi:10.1371/journal.pone.0223456)

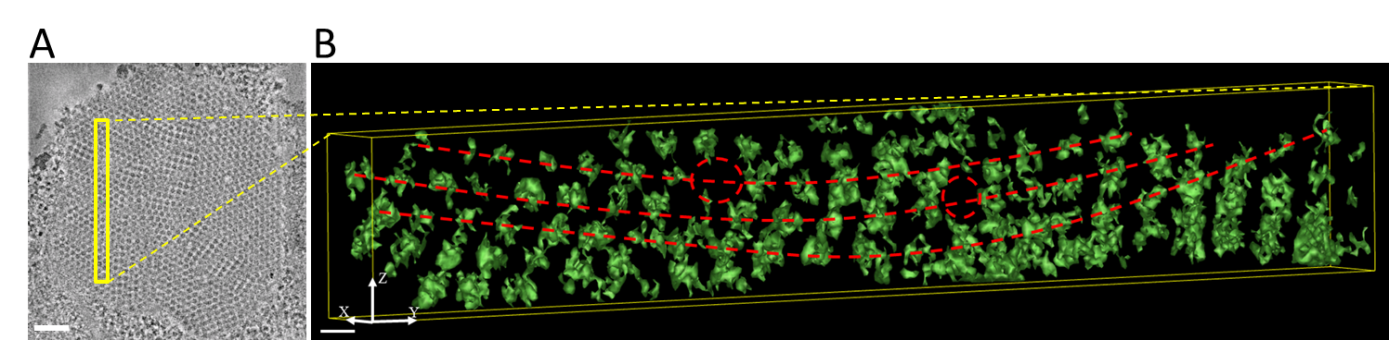

Supplement: S1 Fig — 3D visualization of hIAPP aggregates by performing isosurface segmentation in IMOD. Red dashed lines indicate the bended distribution of hIAPP protein granules in the Z direction. Red dashed circles indicate absence of protein granules. (A) The centre slice from reconstructed tomogram, (B) 50 nm (x) × 650 nm (y) × 150 nm (z) volume. The scale bar in (A) is 100 nm and in (B) is 20 nm. A video was made to show the YZ slices moving from slice No. 300 to No. 1000 along X axis direction in S7 Movie. (TIF) [file pone.0223456.s001.tif]

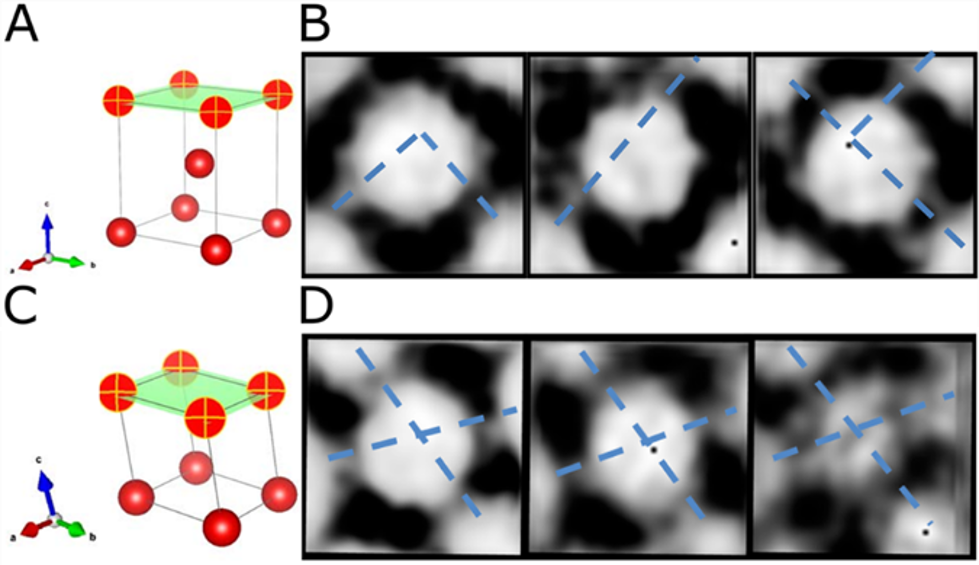

Supplement: S2 Fig — (A) and (C) show the layers parallel to the (001) and the (101) lattice planes that were cut from the BCT unit cell. (B) and (D) are the three averaged classes from the selected particles from (A) and (C). The dashed blue lines indicate the orientations of the linkers between the two nearest protein granules. (TIF) [file pone.0223456.s002.tif]
