# Supplementary material for: 3D analysis of human islet amyloid polypeptide crystalline structures in Drosophila melanogaster
Source: PLoS One. 2019 Oct 10;14(10):e0223456. doi: 10.1371/journal.pone.0223456 (PMC6786548; doi:10.1371/journal.pone.0223456)
Supplement: S1 Data File — (DOCX) [file pone.0223456.s003.docx]

**The binding energy between two nearest protein granules was calculated based on the Hamaker two-body method.**

The estimation of binding energies between two 20 nm globular proteins in the water solution was done using the formula for the binding energy between two spherical particles [1].

$$U\left( r \right)=-\frac{AR_{1}R_{2}}{\left( R_{1}+R_{2} \right)6r}$$

Where A is the Hamaker coefficient, $R_{1} and R_{2}$are the radii of two spheres, $r$ is the edge to edge distance between two spheres. In our case, $R_{1}=R_{2}=10 nm$, $r=5.6 nm.$ The Hamaker coefficient for globular proteins in water was tabulated as $A=2\times{10}^{-20}J\approx0.12 eV$ [2].

$$U\left( r \right)=-\frac{0.12 eV\times10 nm\times10 nm}{\left( 10 nm+10 nm \right)\times6\times5.6 nm} \approx0.02 eV$$

**Reference**

1. Rajagopalan R, Hiemenz, PC. Principles of colloid and surface chemistry. *J Colloid*

*Interface Sci.* 1997;70**:** 234–242.

2. Norde W. Colloids and Interfaces in Life Sciences and Bionanotechnology. New York:

CRC Press 2011.
