# Supplementary material for: 3D analysis of human islet amyloid polypeptide crystalline structures in Drosophila melanogaster
Source: PLoS One. 2019 Oct 10;14(10):e0223456. doi: 10.1371/journal.pone.0223456 (PMC6786548; doi:10.1371/journal.pone.0223456)
Supplement: S1 Discussion — (DOCX) [file pone.0223456.s004.docx]

**Number density of hIAPP granules and the structural imperfections**

The analysis performed on the sub-volumes demonstrates that the hIAPP aggregates have crystalline structures. However, only from the full volume can we see the structure of the protein granules, required to determine the 3D density of the hIAPP granules. In analyzed BCT and triclinic sample volumes, the number density of protein granule is 7.7 × 10^4^/µm^3^ and 5.5 × 10^4^/µm^3^, respectively. By analyzing the structures in all five regions highlighted in Fig. 2C, the average number density of the entire protein aggregate is obtained to be 6.3 × 10^4^/µm^3^.

Although the unit cells BCT and triclinic were identified from sub-volumes, none of them repeated perfectly as a crystal in all three dimensions. From the center slice of the entire reconstructed tomogram in the XY plane (Fig. S1). Clearly, the contrast generated from the hIAPP granules is not visible in several stripe-like areas, a finding not expected to appear in a perfect crystal. To understand the structural imperfection that underlies this contrast change, a volume is extracted from the yellow rectangular area (Fig. S1A) and visualized in the YZ plane (Fig. S1B). All protein granules are crystallized in the triclinic structure, but the protein granules sitting on the (001) lattice planes in the direction of the [010] zone axis are bent in the Z direction (red curved dash line). On some sites of the triclinic structure (indicated by the red dash circles in Fig. S1B), voids or the absences of protein granules were also observed. The bending effect is equally visible in the XZ plane.
